# The Role of Fatty Acid-Binding Protein 4 in Pregnancy Complications and Fetal Health

**DOI:** 10.3390/ijms27219860

**Published:** 2026-11-03

**Authors:** Radzisław Mierzyński, Elżbieta Poniedziałek-Czajkowska, Kamila Świda, Katarzyna Mierzyńska

**Affiliations:** 1 Department of Obstetrics and Perinatology, Medical University of Lublin, ul. Jaczewskiego 8, 20-954 Lublin, Poland; elzbietapc@yahoo.com (E.P.-C.); kamilaswida94@gmail.com (K.Ś.); 2 Medical University of Lublin, Al. Racławickie 1, 20-059 Lublin, Poland

**Keywords:** FABP4, lipid metabolism, obesity, gestational diabetes mellitus, preeclampsia, fetus, trophoblast, recurrent pregnancy loss

## Abstract

Fatty acid-binding protein 4 (FABP4) is an adipokine involved in regulating lipid metabolism, glucose homeostasis, and inflammatory processes. Altered circulating FABP4 concentrations have been associated with insulin resistance, adipose tissue dysfunction, and endothelial abnormalities. Growing evidence suggests that FABP4 dysregulation may contribute to impaired maternal metabolic and vascular adaptation during pregnancy, although the underlying mechanisms remain incompletely understood. FABP4 has been implicated in the pathophysiology of several pregnancy-related conditions, including maternal obesity, gestational diabetes mellitus, hypertensive disorders of pregnancy, preeclampsia, and recurrent pregnancy loss. Circulating FABP4 has also emerged as a potential biomarker for the early identification of women at increased risk of pregnancy-related metabolic and vascular complications, potentially improving risk assessment and providing earlier preventive or therapeutic interventions. This review summarizes current evidence on the role of FABP4 during pregnancy and its potential involvement in the pathophysiology of pregnancy-related complications, with particular emphasis on its biological functions, diagnostic and prognostic potential, and emerging therapeutic implications. Further mechanistic and prospective clinical studies are needed to determine whether FABP4 is a clinically useful biomarker and to clarify its potential as a therapeutic target in the management of pregnancy-related complications.

## 1. Introduction

The metabolic changes that occur throughout pregnancy are modulated, at least in part, by adipokines, which are bioactive mediators derived predominantly from adipose tissue [1]. Adipokines regulate a wide range of metabolic and physiological processes, including pancreatic insulin secretion, insulin sensitivity, adipose tissue distribution, appetite control, inflammatory responses, and systemic energy homeostasis [2,3]. These molecules play an important role in maternal-fetal physiological adaptation and regulate multiple metabolic pathways and processes. Dysregulated metabolic and immune adaptations to pregnancy may contribute to the development of numerous adverse obstetric outcomes, including miscarriage, gestational diabetes mellitus (GDM), preeclampsia (PE), and preterm birth [4]. These complications may increase the risk of long-term maternal morbidity and mortality. Moreover, pregnancy-related metabolic and immune disturbances may also have long-term consequences for offspring health. Alterations in the intrauterine metabolic environment may affect fetal development and increase offspring susceptibility to obesity, insulin resistance, diabetes, and other cardiometabolic disorders later in life, potentially contributing to intergenerational transmission of metabolic risk [5,6].

Fatty acid-binding proteins (FABPs) are a family of low-molecular-weight lipid-binding proteins, approximately 14–15 kDa, that belong to the lipocalin family. FABPs share about 15–70% sequence identity across isoforms [7]. Fatty acid-binding protein 4 (FABP4), also known as adipocyte FABP (A-FABP), was initially identified in adipose tissue and mature adipocytes [8]. It is expressed predominantly in adipocytes and accounts for approximately 1% of the soluble protein content of adipose tissue. FABP4 expression increases markedly during adipocyte differentiation and is transcriptionally regulated by several metabolic and hormonal stimuli, including peroxisome proliferator-activated receptor-γ (PPAR-γ) agonists, fatty acids, dexamethasone, and insulin [9].

Although adipocytes are the primary source of FABP4, the protein is also expressed in immune and vascular cells, including macrophages, dendritic cells, and endothelial cells [10].

FABP4 expression has been detected in endothelial cells of capillaries and small veins in the heart and kidneys. Under physiological conditions, endothelial FABP4 in cardiac capillaries and the interstitial capillary network of skeletal muscle appears to facilitate the transendothelial transport of fatty acids (FAs), thereby supporting their delivery to tissues with high fatty acid metabolic demand [11].

The potential relevance of FABP4 to pregnancy is further supported by evidence demonstrating the expression of FABP family members, including FABP4, in human placental trophoblasts. Given the pivotal role of the placenta in regulating maternal-fetal nutrient exchange, alterations in FABP4-mediated lipid handling may affect placental function and, consequently, fetal growth and metabolic development [12].

Experimental studies increasingly recognize FABP4 as an important regulator of lipid homeostasis; however, the molecular mechanisms underlying its biological effects remain incompletely elucidated [13]. FABP4 may participate in the regulation of nuclear transcriptional processes. The protein can undergo nucleocytoplasmic translocation and may facilitate delivery of fatty acid ligands to nuclear receptors, including members of the peroxisome proliferator-activated receptor (PPAR) family [14].

PPARγ regulates FABP4 expression, suggesting a potential regulatory feedback mechanism. Ayers et al. showed that dynamic nucleocytoplasmic shuttling of FABP4 contributes to PPARγ transcriptional activation [15]. In contrast, FABP4 has been reported to inhibit PPARγ activity in macrophages, highlighting the cell-specific nature of this interaction [16]. These findings underscore the complexity of FABP4 signaling and suggest that its biological effects depend, at least in part, on the cellular context.

In adipocytes, FABP4 also regulates lipolysis through its interaction with hormone-sensitive lipase (HSL) [17]. Experimental studies in FABP4-deficient mice have demonstrated impaired lipolysis both in vitro and in vivo. Notably, when lipolysis was experimentally stimulated, FABP4-deficient animals also exhibited reduced insulin secretion, suggesting a potential link between FABP4-mediated lipid mobilization and pancreatic endocrine function [18]. These observations support the concept that FABP4 may integrate adipose tissue lipid metabolism with systemic metabolic regulation.

Unlike proteins secreted through the conventional secretory pathway, FABP4 lacks an N-terminal signal peptide required for classical secretion [17]. Its release from adipocytes is closely linked to lipolytic activity and is thought to occur via both the adenylyl cyclase–protein kinase A (AC–PKA) and guanylyl cyclase–protein kinase G (GC–PKG) signaling pathways [18]. Thus, FABP4 appears to participate in a dynamic relationship between intracellular lipid mobilization and extracellular signaling. In addition to its regulatory functions, FABP4 may act as an intracellular and extracellular lipid-binding carrier, facilitating the transport of fatty acids released during lipolysis from lipid droplets to sites of metabolic utilization [19].

Secreted FABP4 may also exert direct effects on target cells, extending its potential functions beyond those of an intracellular lipid chaperone to include intercellular coordination of metabolic and inflammatory signaling. Experimental studies have demonstrated diverse biological effects of extracellular FABP4 in several cell types. In cardiomyocytes, FABP4 may impair contractile function, whereas in vascular endothelial cells it has been reported to suppress endothelial nitric oxide synthase (eNOS) expression and/or activity [20]. In the liver, FABP4 promotes hepatic glucose production in both experimental models and in vitro systems [21]. Extracellular FABP4 has also been implicated in regulating vascular smooth muscle cell proliferation and migration and modulating glucose-stimulated insulin secretion by pancreatic β cells [22]. Collectively, these observations suggest that FABP4 may exert pleiotropic effects extending across multiple organs and physiological systems. Consistent with these experimental findings, observational and prospective clinical studies have linked elevated circulating FABP4 concentrations to a broad spectrum of metabolic and cardiovascular disorders, including obesity, insulin resistance, type 2 diabetes mellitus (T2DM), hypertension, cardiac dysfunction, and atherosclerosis [23,24]. The association of FABP4 with disorders characterized by impaired glucose metabolism, chronic inflammation, and vascular dysfunction may be particularly important during pregnancy, during which substantial metabolic and cardiovascular adaptations occur.

**Figure 1:**
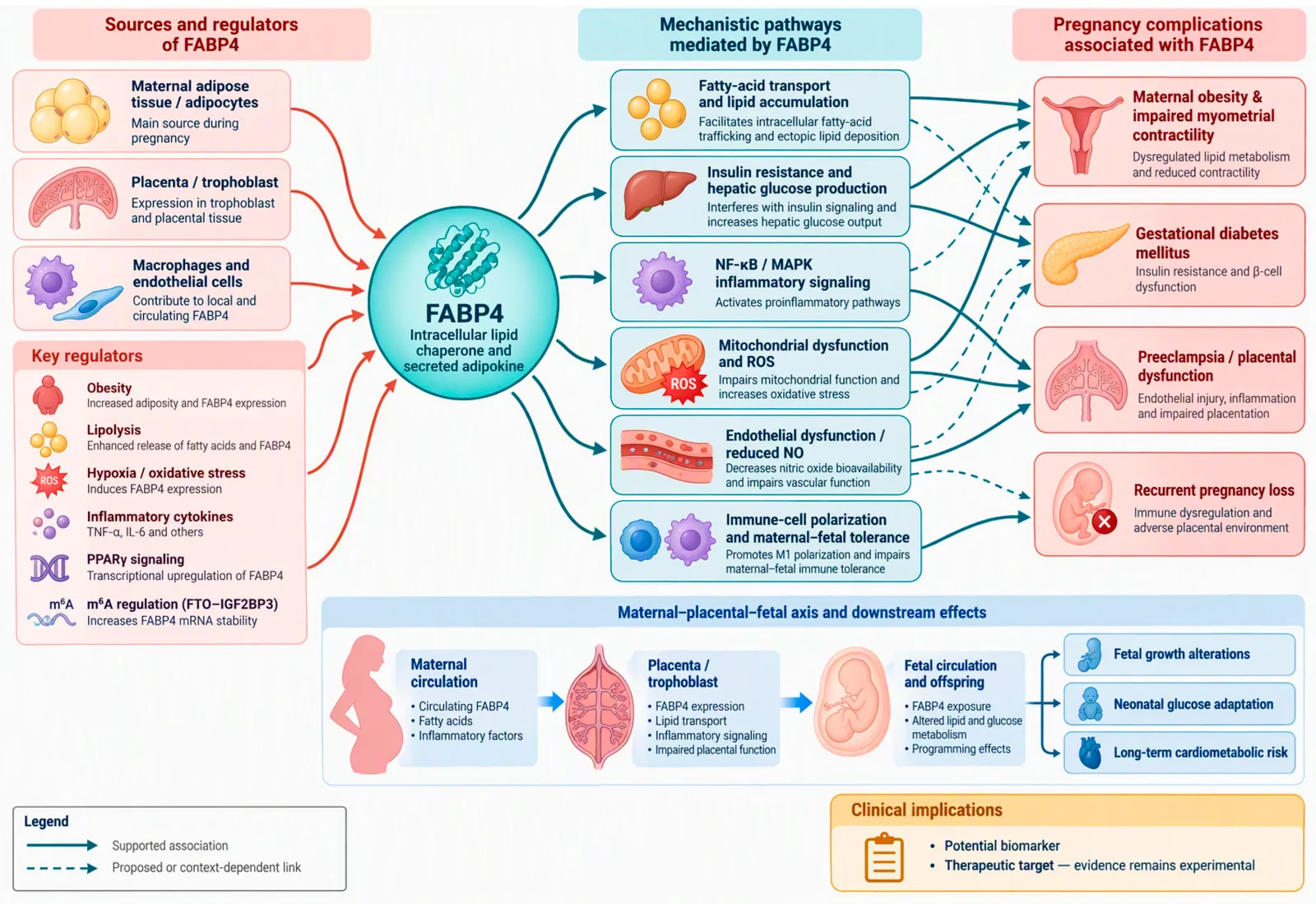
Global mechanistic overview of fatty acid-binding protein 4 (FABP4) in pregnancy. Maternal adipose tissue, placental trophoblasts, macrophages, and endothelial cells contribute to intracellular and circulating FABP4, whose expression and release are modulated by obesity, lipolysis, hypoxia, oxidative stress, inflammatory cytokines, PPARγ signaling, and m6A-dependent regulation. FABP4 may integrate lipid transport, insulin resistance, inflammatory signaling, mitochondrial dysfunction, endothelial impairment, and immune-cell polarization, thereby linking maternal metabolic status with gestational diabetes mellitus, preeclampsia, recurrent pregnancy loss, impaired myometrial contractility, altered fetal growth, neonatal metabolic adaptation, and potential long-term cardiometabolic risk. Solid arrows indicate supported associations; dashed arrows indicate proposed or context-dependent links. FABP4, fatty acid-binding protein 4; FTO, fat mass and obesity-associated protein; IGF2BP3, insulin-like growth factor 2 mRNA-binding protein 3; IL-6, interleukin 6; m6A, N6-methyladenosine; mRNA, messenger RNA; NF-κB, nuclear factor kappa B; NO, nitric oxide; PPARγ, peroxisome proliferator-activated receptor gamma; ROS, reactive oxygen species; TNF-α, tumor necrosis factor alpha.

Emerging evidence also suggests an association between altered FABP4 concentrations and several pregnancy-related complications, including GDM, hypertensive disorders of pregnancy, and recurrent pregnancy loss. However, despite the increasing body of experimental and clinical data, the precise role of FABP4 in the pathophysiology of these conditions remains incompletely defined. It is currently unclear whether altered FABP4 concentrations primarily reflect underlying metabolic and vascular disturbances or whether FABP4 itself contributes to the molecular mechanisms involved in disease development. Figure 1 presents a global mechanistic overview of the role of fatty acid-binding protein 4 (FABP4) in pregnancy.

Despite previous reviews on FABP4’s role in pregnancy complications, we considered it important to revisit this topic in light of growing evidence implicating adipokines, including FABP4, in pregnancy-related complications, as well as the emergence of new studies investigating its role. This review synthesizes the current experimental and clinical evidence on the biological and pathophysiological significance of FABP4 during pregnancy. Particular emphasis is placed on its potential involvement in the development of pregnancy-associated metabolic and vascular complications, including GDM, preeclampsia, and recurrent pregnancy loss. We also discuss the potential implications of altered FABP4 signaling for placental function, fetal development, and long-term offspring health.

This narrative review included a comprehensive search of several major databases, including PubMed, MEDLINE, and ScienceDirect. The databases were searched from the first records until June 2026 using MeSH Terms alone or in combination, such as: “FABP4”, “pregnancy”, “lipid metabolism”, “obesity”, “gestational diabetes mellitus”, “preeclampsia”, “recurrent pregnancy loss”, “fetus” as keywords. All identified publications underwent a detailed and critical assessment, with intentional emphasis on studies most relevant to the primary topic. The references included in the selected publications were also analyzed to find additional relevant publications.

## 2. The Role of FABP4 in Pregnancy Complications

### 2.1. Maternal Obesity

Maternal overweight and obesity during pregnancy are independently correlated with an increased risk of a broad spectrum of adverse obstetric and neonatal outcomes, irrespective of pre-existing chronic comorbidities. These complications include hypertensive disorders of pregnancy, particularly gestational hypertension and PE, GDM, cesarean delivery, and labor induction [25]. An increasing maternal body mass index (BMI) is also associated with a higher incidence of adverse neonatal outcomes, including very preterm birth before 32 weeks of gestation, fetal macrosomia, transient tachypnea of the newborn, neonatal sepsis, and admission to a neonatal intensive care unit (NICU) [25,26]. Maternal overweight and obesity are also recognized as important determinants of long-term metabolic health in the offspring. Excessive adipose tissue development and lipid accumulation during fetal life may increase susceptibility to childhood obesity and contribute to subsequent development of metabolic syndrome and cardiovascular disease [27].

Circulating FABP4 concentrations have consistently been linked to obesity-associated metabolic parameters, including serum triglycerides, cholesterol, and leptin, in both human and animal studies [28]. Obese patients have been shown to exhibit significantly higher circulating FABP4 concentrations than lean individuals, with FABP4 levels demonstrating positive associations with waist circumference, blood pressure, and the degree of insulin resistance [23]. Visceral adipose tissue exhibits greater lipolytic activity than subcutaneous adipose tissue, while excessive visceral fat accumulation is associated with increased oxidative stress. In a prospective clinical study, Saito et al. demonstrated that oxidative modifications may induce conformational changes in FABP4, potentially altering its ligand-binding properties and increasing its affinity for saturated fatty acids relative to essential polyunsaturated fatty acids, including linoleic acid and α-linolenic acid [29].

Moreover, baseline FABP4 concentrations have been identified as an independent predictor of incident metabolic syndrome over a five-year follow-up period in a Chinese cohort [23]. Similarly, a 10-year prospective study found that elevated baseline FABP4 levels independently predicted subsequent development of T2DM [30]. Genetic studies in animals suggest that allelic variation within the FABP4 gene region, which is associated with reduced FABP4 expression in adipose tissue, may be linked to a lower susceptibility to cardiovascular disease [31].

Recent clinical studies have suggested that FABP4 may have potential clinical utility as a biomarker of obesity and an early indicator of obesity-related metabolic and renal complications, including T2DM and diabetic nephropathy [32,33]. Other clinical studies have shown that circulating FABP4 concentrations increase during pregnancy and are associated with gestational weight gain; they also indicate a positive relationship between circulating FABP4 concentrations and maternal BMI [2], particularly among women with a BMI above 25 kg/m^2^ [34].

The potential pathophysiological relevance of FABP4 is further supported by experimental studies investigating its role in glucose and lipid metabolism. In leptin-deficient mice (*ob*/*ob* mice), which develop obesity and metabolic disturbances, pharmacological inhibition of FABP4 with the small-molecule inhibitor BMS309403 improved insulin sensitivity. Similarly, mice in which obesity was induced by a high-fat diet (diet-induced obesity, DIO) were less susceptible to obesity-related insulin resistance and T2DM development when FABP4 was genetically deleted [35]. A novel series of FABP4/5 dual inhibitors represented by Compounds **1**–**3**, compared with BMS309403, had significant in vitro potency toward both FABP4 [36]. When chronically administered to DIO mice, BMS309403 and Compound **3** reduced plasma triglyceride and free FA concentrations [37].

In an experimental model of obesity, dual gene silencing of FABP4/5 in white adipocytes using an adipocyte-targeting peptide was associated with substantial reductions in body weight and inflammatory activity, together with improved insulin sensitivity [38]. Collectively, these findings suggest an important role for FABP4 in the regulation of lipid metabolism, insulin sensitivity, inflammation, and adiposity.

The potential involvement of FABP4 in obesity-related metabolic dysfunction may also be relevant to uterine physiology and the mechanisms underlying labor. Women with obesity are at increased risk of inefficient uterine contractility during labor, and spontaneous contractions of myometrial strips obtained from obese patients decrease with increasing body weight [39].

Clinical and experimental observations suggest that endocrine, metabolic, and mechanical mechanisms regulate myocyte contractile activity, which depends critically on adequate cellular energy metabolism and mitochondrial function.

Excessive intracellular lipid accumulation and increased fatty acid availability may impair mitochondrial function and, consequently, compromise smooth muscle contractility and cellular metabolic homeostasis [39]. In obesity, mitochondrial physiology is altered, including impairment of the electron transport chain and dysregulation of genes involved in oxidative metabolism and mitochondrial biogenesis. Among the key molecular regulators implicated in these processes are PPAR-γ and peroxisome proliferator-activated receptor gamma coactivator 1-alpha (PGC-1α), which play central roles in maintaining mitochondrial function and energy metabolism [39,40].

In this context, emerging evidence suggests that FABP4 may contribute directly to obesity-related myometrial dysfunction. In experimental studies, upregulation of FABP4 has been associated with excessive lipid accumulation, impaired mitochondrial function, and reduced myometrial contractility [40]. Mechanistically, elevated FABP4 levels appeared to compromise mitochondria-associated membrane (MAM) integrity, thereby interfering with fatty acid utilization and reducing cellular ATP generation. Increased FABP4 expression has been detected in myometrial tissue obtained from obese pregnant women, in the myometrium of mice on a high-fat diet (HFD), and in primary human uterine smooth muscle cells (USMCs) exposed to palmitic acid (PA) [40]. Notably, siRNA-mediated suppression of FABP4 partially restored the contractile capacity of dysfunctional myometrium [40].

Collectively, these findings suggest that FABP4 may play a role in obesity-associated myometrial dysfunction [40]. However, these observations are preliminary, have not yet demonstrated clinical applicability, and require further investigation.

### 2.2. Gestational Diabetes Mellitus

Gestational diabetes mellitus is a common metabolic complication of pregnancy, affecting approximately 14% of pregnancies worldwide. It is defined as glucose intolerance that is first recognized or diagnosed during pregnancy [41]. GDM is associated with an increased risk of adverse maternal, fetal, and neonatal outcomes and is considered an important risk factor for the subsequent development of metabolic disorders in both mothers and their offspring. Although the pathophysiology of GDM is multifactorial, a growing body of evidence suggests that dysregulation of adipokine metabolism, including adiponectin, FABP4, leptin, nesfatin, vaspin, and chemerin, may contribute to the development of insulin resistance and impaired glucose homeostasis during pregnancy [42,43].

FABP4 is a potential candidate involved in the pathophysiology of GDM [2]. In clinical studies, increased circulating FABP4 concentrations observed in the maternal serum of patients with GDM may reflect enhanced FABP4 production by both adipose and placental tissues [44,45,46]. In women with GDM, placental and decidual FABP4 mRNA expression has been reported to be higher than in corresponding tissues from uncomplicated pregnancies [46]. FABP4 derived from the placental and adipose tissue may exert complementary metabolic and pro-inflammatory effects, potentially contributing to impaired insulin signaling. Circulating FABP4 concentrations decrease significantly after an oral glucose tolerance test and after consuming a high-fat meal, concomitant with an increase in circulating insulin levels.

This postprandial decrease in plasma FABP4 may be associated, at least in part, with insulin-mediated antilipolytic signaling, which suppresses FABP4 secretion from adipose tissue [47].

Although the mechanisms underlying the relationship between FABP4 and insulin sensitivity remain incompletely elucidated, FABP4 may contribute to impaired insulin responsiveness by modulating pro-inflammatory signaling pathways, including the mitogen-activated protein kinase (MAPK) pathway. Experimental studies show that FABP4 deficiency in macrophages may enhance glucose uptake and improve insulin signaling in adipocytes [48]. Moreover, obesity is associated with macrophage polarization toward a pro-inflammatory M1 phenotype, characterized by increased production and secretion of cytokines such as tumor necrosis factor-α (TNF-α) and interleukin-6 (IL-6) [48].

Furuhashi et al. suggested that pro-inflammatory mediators, including TNF-α and IL-6, can enhance FABP4 expression in adipose tissue. The proposed mechanism involves activation of the nuclear factor kappa B (NF-κB) signaling pathway, which may subsequently promote FABP4 gene transcription [49].

These findings suggest that FABP4 may contribute to obesity-associated insulin resistance, at least in part, through interactions between lipid metabolism, macrophage activation, and inflammatory signaling [50].

The relationship between FABP4 and insulin sensitivity may also involve PPARγ, a key transcriptional regulator of adipocyte differentiation and metabolic function. PPARγ regulates FABP4 expression at the transcriptional level and may therefore indirectly contribute to the regulation of insulin sensitivity [51]. It is highly expressed in mature adipocytes and controls the expression of numerous genes involved in adipogenesis and lipid metabolism. Although PPARγ is best known for its role in adipocyte differentiation and function, its metabolic effects extend beyond adipose tissue. Evidence from liver-specific knockout models further suggests that PPARγ may contribute to the regulation of systemic glucose homeostasis and insulin sensitivity. However, these findings should be interpreted in a tissue-specific context and do not directly establish the role of PPARγ in adipocytes [52,53]. Conversely, FABP4 may modulate its activity and protein abundance [50]. Animal studies have shown that, in addition to facilitating the delivery of endogenous ligands to PPARγ, FABP4 may promote the ubiquitination and subsequent proteasomal degradation of this nuclear receptor, potentially limiting PPARγ-dependent transcriptional activity [54,55]. Consistent with this proposed regulatory mechanism, FABP4-deficient preadipocytes exhibit enhanced adipogenic differentiation, suggesting that FABP4 may influence adipogenesis and insulin responsiveness, at least in part, by modulating PPARγ signaling [54,56]. Conversely, pharmacological activation of PPARγ with selective agonists increases PPARγ-dependent transcriptional activity. It improves insulin sensitivity, further supporting a functional relationship between FABP4 and PPARγ in regulating adipocyte biology and systemic metabolic homeostasis [57]. Furthermore, experimental findings suggest that FABP4 may exert insulinotropic effects, with some evidence indicating functional similarities to the insulin-secretory actions of glucagon-like peptide-1 (GLP-1) [56].

Clinical evidence further supports an association between circulating FABP4 concentrations and the metabolic phenotype of GDM. Most clinical studies have reported significantly higher serum FABP4 concentrations in women with GDM compared with women with normoglycemic pregnancies [58,59,60,61]. Additionally, in GDM patients, circulating FABP4 concentrations have been positively associated with several metabolic and obstetric parameters, including fasting glucose and insulin concentrations, serum triglyceride concentrations, and neonatal birth weight [60]. These associations may be influenced, at least in part, by maternal adiposity and the metabolic alterations accompanying increased adipose tissue mass. Evidence further supports this concept by implicating FABP4 in promoting lipid accumulation and inhibiting fatty acid β-oxidation [46]. FABP4 overexpression has also been observed in visceral white adipose tissue from women with GDM, where FABP4 may act as a coactivator of glucagon-mediated hepatic gluconeogenesis [62].

Moreover, FABP4 has been reported to be more strongly associated with GDM risk than other adipokines, including leptin and retinol-binding protein 4 (RBP4) [63]. These observations further support the potential clinical utility of circulating FABP4 as a predictive biomarker for GDM, consistent with previously reported evidence [60].

Importantly, alterations in circulating FABP4 concentrations may occur before the clinical diagnosis of GDM, suggesting a potential role for FABP4 as an early biomarker of disease risk. GDM is typically diagnosed in the second or third trimester, when metabolic disturbances may have already adversely affected maternal and fetal health. Therefore, the identification of reliable biomarkers in early pregnancy could facilitate early risk stratification and enable timely interventions, potentially reducing adverse maternal and neonatal outcomes and improving the long-term health of both mother and offspring. Zhang et al. reported a marked increase in circulating FABP4 concentrations among women with GDM between the second and third trimesters [63]. Jin et al. found that women with FABP4 concentrations in the highest tertile during the first and second trimesters had a 5.3% and 44.7% higher risk of developing GDM, respectively, compared with women in the lowest tertile [64]. However, these preliminary findings, based on a small patient cohort, require further validation in larger study populations, accounting for potential confounding factors such as maternal obesity, insulin resistance, and metabolic status.

### 2.3. Preeclampsia

Preeclampsia is a progressive multisystem disorder of pregnancy characterized by the new onset of hypertension after 20 weeks of gestation, accompanied by proteinuria and/or evidence of maternal end-organ dysfunction in a previously normotensive patient. The condition may also occur postpartum. Preeclampsia remains a major cause of maternal and perinatal morbidity and mortality and is strongly associated with adverse pregnancy outcomes, including preterm birth, fetal growth restriction (FGR0, and perinatal death [65]. A systematic review by Abalos et al. estimates the global prevalence of preeclampsia at 4.6% (95% CI, 2.7–8.2%), although substantial geographic variation has been reported [66]. The pathogenesis of PE is complex and heterogeneous, involving multiple pathophysiological mechanisms. Placental dysfunction is considered a central component of the disease process in both early- and late-onset PE and may subsequently contribute to maternal endothelial and vascular abnormalities.

An increasing body of evidence suggests that FABP4 may contribute to the pathophysiology of PE. Several clinical studies have reported an association between elevated circulating FABP4 concentrations and the development of hypertension and PE during pregnancy [67,68,69,70]. Moreover, maternal serum FABP4 concentrations have been reported to be significantly higher in women with severe PE than in those with mild PE, suggesting a possible relationship between circulating FABP4 levels and disease severity [67]. Although these findings support an association between FABP4 and PE, the exact mechanisms underlying this relationship remain incompletely understood.

Placental hypoxia and impaired placental perfusion are key early pathological mechanisms in PE and are often accompanied by alterations in lipid metabolism, including elevated circulating fatty acid and triglyceride concentrations [70]. Scifres et al. demonstrated the presence of FABP4 in placental tissue and trophoblastic cells, suggesting that dysregulation of FABP4 expression or activity may contribute to the development of pregnancy-related disorders [68].

Recent animal studies have provided further evidence for a potential role of FABP4 in placental dysfunction. In a rat model of PE, placental tissue exhibited increased FABP4 expression.

Elevated FABP4 expression in trophoblasts was associated with increased production of pro-inflammatory cytokines, including IL-6 and TNF-α, as well as intracellular lipid accumulation [71]. These observations suggest that FABP4 dysregulation may influence both inflammatory signaling and lipid homeostasis within the placenta, potentially contributing to trophoblast dysfunction. Experimental and cohort studies further link increased FABP4 expression to trophoblast lipid metabolism, mitochondrial function, and oxidative stress [72]. Together, these findings suggest that FABP4 may represent a mechanistic link between impaired fatty acid metabolism and mitochondrial dysfunction in trophoblasts, which could contribute to placental insufficiency in PE [72].

Although FABP4 expression is markedly increased in preeclamptic placentas, pharmacological inhibition or siRNA-mediated silencing of FABP4 has been reported to reduce trophoblast proliferation and impair mitochondrial function. Sun et al. proposed that FABP4 upregulation in PE may represent a compensatory response to placental stress [72]. Under physiological conditions, FABP4 may support trophoblast growth by facilitating fatty acid transport and ensuring the availability of fatty acids as metabolic substrates. During the early stages of placental ischemia and hypoxia, moderate FABP4 upregulation could therefore constitute an adaptive response aimed at maintaining trophoblast metabolic activity and cellular viability. However, persistent ischemic and hypoxic stress accompanying disease progression may lead to sustained and excessive FABP4 expression. Beyond a potentially compensatory range, this dysregulation may contribute to lipid accumulation, oxidative stress, and mitochondrial dysfunction, thereby exacerbating placental disorder [72]. In PE, FABP4 overexpression may promote intracellular FFA accumulation, potentially exceeding mitochondrial oxidative capacity and exacerbating oxidative stress and trophoblast dysfunction. This context-dependent effect may help explain the dual role of FABP4, supporting physiological trophoblast growth under normal conditions while contributing to placental pathology when excessively expressed in PE. These observations are consistent with previous findings in adipocytes, where physiological FABP4 activity supports lipid homeostasis, whereas overexpression has been linked to metabolic inflammation. The same concept may extend to trophoblast dysfunction in PE, although further research is needed to establish this relationship [72].

In addition to its potential role in the placenta, circulating FABP4 may contribute to the systemic metabolic and inflammatory abnormalities associated with PE. In a prospective clinical study, Scifres et al. suggested that increased maternal serum FABP4 concentrations may contribute to PE pathogenesis through mechanisms linked to inflammation, insulin resistance, and inappropriate lipid metabolism [68]. FABP4 has also been implicated in proatherogenic processes and macrophage lipid handling, including alterations in cholesterol metabolism. Experimental evidence indicates that FABP4 may influence macrophage inflammatory signaling and transcriptional activity, thereby potentially affecting the production of pro-inflammatory mediators such as TNF-α [68,73]. These mechanisms may be particularly relevant to PE, in which systemic inflammation, metabolic disturbances, and vascular dysfunction are closely associated.

Another potential mechanism linking FABP4 to PE pathophysiology involves vascular endothelial dysfunction. Impaired vascular reactivity and endothelial dysfunction are characteristic features of PE, and abnormalities in endothelial function may precede clinical manifestation of the disease. Although direct evidence linking FABP4 to endothelial dysfunction in PE remains limited, clinical studies conducted in other populations provide biologically relevant evidence. In patients with T2DM, circulating FABP4 concentrations have been inversely associated with endothelial function, suggesting a potential link between higher FABP4 levels and vascular endothelial impairment [74]. Furthermore, elevated serum FABP4 concentrations have been identified as an independent predictor of vascular inflammation in nondiabetic individuals. Using [18F]-fluorodeoxyglucose positron emission tomography, Yoo et al. demonstrated an association between circulating FABP4 levels and vascular inflammatory activity, suggesting that FABP4 may be involved in pathways linking altered lipid metabolism to vascular inflammation [75].

Further evidence of a possible association between FABP4 and PE has been obtained in pregnancies complicated by type 1 diabetes mellitus (T1DM), a population at increased risk of developing PE. In a prospective clinical study by Wotherspoon et al., women with T1DM who subsequently developed PE had significantly higher serum FABP4 concentrations than those who did not. Notably, increased FABP4 concentrations were observed both at randomization and at 26 weeks of gestation, preceding the clinical diagnosis of PE [76]. Similarly, a prospective longitudinal cohort study found that women with T1DM who subsequently developed PE had higher circulating FABP4 concentrations throughout pregnancy than those who did not develop the disorder [77]. The observation that elevated FABP4 levels may precede the clinical onset of PE suggests that FABP4 could be involved in early pathophysiological processes or serve as a potential biomarker of PE development in this high-risk population [76].

However, other factors may affect these findings and should be considered. Preeclampsia is closely associated with maternal obesity, insulin resistance, and GDM, all of which may independently influence circulating FABP4 concentrations. Consequently, although the available evidence supports an association between increased FABP4 levels and PE, it remains unclear whether FABP4 represents an independent pathogenic factor, a compensatory response to placental and metabolic stress, or a biomarker reflecting the underlying metabolic and vascular disturbances associated with the disease. Further prospective and mechanistic studies are therefore needed to clarify the precise role of FABP4 in the development and progression of preeclampsia.

### 2.4. Recurrent Pregnancy Loss

Recurrent pregnancy loss (RPL) is generally defined as the occurrence of two or more spontaneous abortions and affects approximately 1–5% of couples. Despite advances in diagnostic evaluation, a significant part of cases remains unexplained and is classified as idiopathic [78]. Disturbances in immunological regulation, placental development, trophoblast function, and cellular metabolism may contribute to the pathophysiology of RPL [78].

A growing body of evidence, derived mainly from experimental and non-clinical studies, suggests that FABP4 may also be involved in processes essential for early pregnancy, including embryo implantation, maternal immune adaptation, and trophoblast function. Within the placenta, FABP4 has been identified in endothelial cells [12] as well as in endometrial epithelial cells [79], suggesting that its expression may be relevant to regulating the maternal-fetal interface. Through its involvement in lipid metabolism and cellular signaling, FABP4 may help maintain placental function during pregnancy [80].

FABP4 expression has also been detected in several immune cell populations at the maternal-fetal interface, including macrophages [81], dendritic cells [82,83], and natural killer (NK) cells [83], which are crucial in the immunological reactions during embryo implantation. Elevated FABP4 levels in macrophages moderate polarization by promoting the M1 pro-inflammatory phenotype, while reduced FABP4 levels favor the M2 anti-inflammatory phenotype [84,85]. FABP4 has been proposed to modulate macrophage activation through the Janus kinase 2/signal transducer and activator of transcription 2 (JAK2/STAT2) signaling pathway. Conversely, FABP4 deficiency in macrophages has been associated with alterations in sirtuin 3 (SIRT3)-dependent signaling and increased production of reactive oxygen species (ROS) [86]. FABP4 may also participate in regulating NK-cell activity. Increased FABP4 expression has been associated with reduced interferon (IFN) production by NK cells, which may contribute to the establishment and maintenance of maternal immune tolerance during pregnancy [87]. Dysregulation of FABP4-dependent signaling may disturb immune homeostasis at the maternal-embryonic interface, potentially affecting implantation and increasing the risk of pregnancy loss [87].

Clinical evidence also suggests an association between FABP4 and pregnancy loss. In a clinical study by Yang et al., patients with RPL (at 8–12 weeks of gestation) had higher FABP4 levels compared to healthy pregnant women [88]. These findings suggest that FABP4 may have potential as a biomarker of early pregnancy complications; however, further studies are required to determine its diagnostic and prognostic value. Interestingly, there was no association between placental FABP4 expression at both the gene and protein levels and its circulating concentration. The authors suggest this may be attributable to trophoblast sampling at approximately 9 weeks of gestation, when FABP4 expression remains relatively low [88].

FABP4 may also contribute to normal placental development and placentation later in gestation [89]. In an animal study, Roberti et al. demonstrated that pharmacological inhibition of the mammalian target of rapamycin (mTOR) signaling pathway was associated with embryonic resorption and reduced FABP4 expression in decidual cells [90]. Furthermore, inhibiting PPAR signaling was associated with impaired placentation and reduced FABP4 expression. These observations suggest that FABP4 may participate in the molecular mechanisms regulating decidualization and placental development [90].

More recent evidence suggests a potential relationship between dysregulated FABP4 expression, alterations in N6-methyladenosine (m6A) RNA modification, and the occurrence of spontaneous abortion [23]. N6-methyladenosine is the most prevalent internal post-transcriptional modification of messenger RNA (mRNA) and is an important regulator of RNA metabolism and gene expression. This modification is particularly enriched around stop codons and within untranslated regions, including the 3′ untranslated region (3′-UTR), and is commonly associated with the conserved RRACH sequence motif [91].

The regulation of m6A modification is mediated by three major groups of proteins: methyltransferases, which function as “writers”; demethylases, primarily fat mass and obesity-associated protein (FTO) and alkB homolog 5 (ALKBH5), which act as “erasers”; and m6A-binding proteins, such as members of the insulin-like growth factor 2 mRNA-binding protein (IGF2BP), which act as “readers” [92].

The m6A modification regulates multiple aspects of post-transcriptional gene expression, including mRNA stability, translation, degradation, and cellular differentiation. Dysregulation of the m6A regulatory system has been implicated in several reproductive and gynecological disorders, including infertility [93], ovarian cancer [94], and endometriosis [95]. Furthermore, alterations in m6A methylation have been reported in endometrial tissue from patients with RPL, suggesting that disturbances in epitranscriptomic regulation may contribute to the pathophysiology of this condition [91,95].

Molecular analyses showed that alterations in m6A RNA methylation regulators, particularly fat mass and obesity-associated protein (FTO) and insulin-like growth factor 2 mRNA-binding protein 3 (IGF2BP3), may be involved in the suppression of FABP4 expression in patients with RPL [96]. A significant increase in global m6A levels in chorionic villous cells obtained from patients with RPL was also observed. Additionally, the expression of the m6A demethylase fat mass and FTO was markedly reduced [97]. Decreased FTO levels downregulated FABP4, whereas higher FTO levels upregulated FABP4 mRNA. Similarly, suppression of the m6A reader IGF2BP3 decreased FABP4 mRNA levels [91].

A potential association between FABP4 expression and trophoblast mitochondrial function has also been identified. FABP4 knockdown reduced the oxygen consumption rate of trophoblastic cells, accompanied by decreased ATP production, basal respiration, and spare respiratory capacity. This indicates that reduced FABP4 expression may impair mitochondrial oxidative metabolism in trophoblast cells [93].

Collectively, these observations suggest that the m6A-FTO-IGF2BP3 regulatory axis may represent an important epitranscriptomic mechanism involved in controlling gene expression during pregnancy [98]. Through the activity of the FTO demethylase and m6A-binding proteins such as IGF2BP3, this pathway may influence RNA stability and translation, thereby providing an additional layer of gene regulation during placental development [98]. The observed relationship between FTO, IGF2BP3, and FABP4 expression further suggests that m6A-dependent regulation may contribute to the maintenance of trophoblast function, including mitochondrial oxidative metabolism [91]. Alterations in this regulatory network could potentially affect the expression of genes involved in trophoblast differentiation, placental growth, nutrient transport, and the maintenance of the maternal-fetal interface [98]. Furthermore, changes in m6A-dependent RNA regulation may influence placental responses to metabolic, inflammatory, or hypoxic stress, although the precise contribution of the FTO-IGF2BP3 axis to these processes remains to be established.

Investigating the activity of this pathway at different stages of pregnancy may therefore help clarify the role of post-transcriptional regulation in placental adaptation and the development of pregnancy-related complications. Dai et al. showed significantly reduced FABP4 expression in chorionic villous cells from patients with RPL at 6–9 weeks of gestation (slightly earlier than in the Yang study) compared with controls. They also observed a concomitant reduction in FABP4 and superoxide dismutase 2 (SOD2) expression and suggested that altered FABP4 regulation may impair lipid homeostasis in trophoblasts, with concomitant attenuation of mitochondrial oxidative phosphorylation, increased oxidative stress, and reduced SOD2 expression [91].

Alterations in lipid metabolism and mitochondrial oxidative phosphorylation may consequently increase oxidative stress and adversely affect trophoblast function. Such metabolic disturbances could contribute to impaired placental development and may be involved in the pathogenesis of pregnancy loss [93].

In addition to its potential role in RPL, FABP4 may also be involved in impaired fertility. An association between FABP4 and polycystic ovary syndrome (PCOS), a common endocrine disorder associated with reproductive dysfunction, has been observed. In animal studies, FABP4 expression has also been observed in apoptotic granulosa cells within atretic antral follicles, suggesting a potential involvement in ovarian pathophysiology. This finding may be particularly relevant to PCOS, a disorder frequently associated with insulin resistance [99]. FABP4 mRNA expression in isolated granulosa cells has been reported to be significantly higher in women with PCOS than in unaffected controls. Furthermore, genetic studies suggest a potential association between specific FABP4 gene polymorphisms and susceptibility to PCOS [100].

Overall, FABP4 appears to play a role in early pregnancy, particularly in establishing maternal immunological tolerance and regulating appropriate trophoblast invasion. However, it should be strongly emphasized that these assumptions are based primarily on experimental studies. Further studies, particularly well-designed in vivo investigations, are required to clarify its biological function and assess its potential clinical applicability.

## 3. The Impact of FABP4 on Fetal and Offspring’s Health

During pregnancy, maternal-fetal metabolic adaptations substantially alter glucose and lipid utilization to ensure an adequate supply of energy substrates for fetal growth and development. Although endogenous glucose production by the fetus is largely suppressed in utero, hepatic glucose production is rapidly activated after birth [62].

Physiological pregnancy is characterized by progressive metabolic adaptations that promote the preferential lipid utilization as an energy substrate, thereby preserving glucose availability for fetal growth and development [101]. These changes become more pronounced in late pregnancy and are part of the normal metabolic adaptations that occur during pregnancy. However, maternal metabolic disorders, including obesity and GDM, may disrupt these physiological adaptations and modify the intrauterine nutrient environment. Consequently, offspring exposed to maternal obesity or GDM may develop alterations in carbohydrate and lipid metabolism that can persist beyond the neonatal period and contribute to long-term metabolic programming [102]. The consequences of abnormal fetal growth may extend beyond the perinatal period. Fetal growth restriction has been associated with an increased risk of cardiovascular disease and other chronic metabolic disorders later in life [103]. Conversely, excessive fetal growth and elevated birth weight have also been linked to an increased susceptibility to obesity, cardiovascular disease, and T2DM in adulthood. These observations suggest that both insufficient and excessive fetal growth may be associated with adverse long-term cardiometabolic outcomes [104].

During the neonatal period, FABP4 has been implicated in regulating fatty acid metabolism and may play an important role in maintaining glucose homeostasis during the metabolic transition after delivery [62,105]. Changes in its expression or activity may contribute to the link between maternal metabolic disturbances and metabolic changes in the offspring [103].

Clinical evidence supporting a potential maternal-fetal relationship in FABP4 metabolism has emerged from studies measuring circulating FABP4 concentrations in maternal and umbilical cord blood [106]. Patro-Małysza et al. reported significantly higher FABP4 concentrations in umbilical cord serum obtained from neonates born to mothers with GDM. Cord blood FABP4 concentrations were positively correlated with maternal serum FABP4 levels, and multiple linear regression analysis demonstrated a significant association between maternal and cord blood FABP4 concentrations [107]. These findings suggest that maternal metabolic status may be reflected, at least in part, in fetal circulating FABP4 concentrations. Nevertheless, the authors emphasized that maternal circulating factors may influence FABP4 levels in umbilical cord blood, highlighting the need to further investigate the relative contributions of maternal, placental, and fetal determinants of circulating FABP4.

A significant association between maternal circulating FABP4 concentrations and offspring FABP4 levels has also been reported, providing additional evidence for a potential relationship between maternal and fetal FABP4 status [60]. However, these observations do not necessarily indicate evidence of direct transplacental transfer or a causal effect of maternal FABP4 on fetal metabolic programming. Placental production, fetal tissue expression, and other maternal and fetal metabolic factors may also influence fetal circulating FABP4 concentration.

Association between maternal and fetal cord blood levels is observed not only in GDM patients but also in those with PE. In a clinical study, Yan et al. reported significantly lower FABP4 concentrations in umbilical cord blood from pregnancies complicated by both early- and late-onset PE compared with controls delivered after 34 weeks of gestation. Interestingly, cord blood FABP4 concentrations were inversely associated with maternal serum FABP4 concentrations and placental FABP4 mRNA expression [108]. These observations suggest that maternal, placental, and fetal FABP4 compartments may be regulated independently and that the relationship between circulating FABP4 concentrations and placental FABP4 expression may differ according to the pathophysiological context.

The placenta may also serve as an additional source and functional site of FABP4 activity during gestation. In an animal study, Makkar et al. showed that perinatal hypoxia selectively increased the expression of FABP1, FABP3, and FABP4 in placental trophoblasts, whereas fatty acid exposure further enhanced FABP4 expression [12]. Given the established role of FABP4 in intracellular fatty acid transport, these findings raise the possibility that FABP4 contributes to fatty acid handling in trophoblastic cells. In a prospective clinical study, Ciborowski et al. identified associations between neonatal birth weight and maternal circulating concentrations of selected lipid species and FABP4, suggesting a potential relationship between maternal lipid metabolism, FABP4 availability, and fetal growth [109]. Elevated maternal FABP4 concentrations have also been associated with an increased risk of fetal macrosomia, defined as a birth weight greater than 4000 g [110]. Conversely, elevated maternal circulating FABP4 concentrations have been associated with an increased risk of FGR [111]. In a clinical study involving 83 pregnant women, Bolluk et al. reported significantly higher median maternal serum FABP4 concentrations in pregnancies complicated by FGR than in uncomplicated controls. Maternal FABP4 concentrations were also higher in pregnancies with abnormal fetal Doppler flow than in those with normal Doppler flow patterns, suggesting a potential association between elevated FABP4 levels and altered fetoplacental hemodynamics [111]. However, fetal growth outcomes are multifactorial and depend on maternal metabolic status, placental function, and pregnancy complications, such as PE. The reported associations suggest that the relationship between FABP4 and fetal growth is complex and may vary with maternal factors, placental function, and fetal growth phenotype.

In addition to its potential association with fetal growth, FABP4 may also contribute to neonatal glucose homeostasis. Experimental evidence suggests that FABP4 may promote hyperglycemia by enhancing hepatic glucose production through the regulation of key gluconeogenic enzymes [112]. Notably, circulating FABP4 concentrations have been reported to be higher in the fetal than in the maternal circulation and to increase approximately threefold during the first hours after birth [62]. This postnatal increase coincides with the initiation of endogenous glucose production and the metabolic transition from placental nutrient supply to autonomous energy metabolism, suggesting that FABP4 may contribute to this adaptive process [62].

In neonates, circulating FABP4 concentrations have been reported to be inversely associated with blood glucose concentrations, with the highest FABP4 levels observed among infants with hypoglycemia. A nonsignificant trend toward lower FABP4 concentrations has also been reported in large-for-gestational-age (LGA) neonates [112]. One possible explanation is that fetal hyperinsulinemia, which may accompany excessive fetal growth, could influence circulating FABP4 concentrations. However, this hypothesis remains unconfirmed, and the mechanisms underlying this association require further investigation. Similarly, researchers have reported an inverse association between birth weight and umbilical cord FABP4 concentrations in term neonates [107]. In contrast, preterm infants who were small for gestational age (SGA) showed slightly lower FABP4 concentrations [113]. Thus, these observations suggest that circulating FABP4 levels may be influenced by gestational age, fetal growth, glucose homeostasis, and the metabolic characteristics of the intrauterine environment.

FABP4 may also interact functionally with glucagon signaling, potentially enhancing glucagon-induced glycogenolysis and the expression of glucagon-responsive genes while acting as an endocrine counter-regulatory signal to insulin [114]. The marked increase in circulating FABP4 concentrations after birth may therefore represent a part of the coordinated hormonal and metabolic adaptation to the abrupt shift in energy metabolism at birth. With increased glucagon, catecholamines, and cortisol, adipocyte-derived FABP4 may in part facilitate glucagon activity and promote the availability of free fatty acids as an alternative energy substrate for the liver during the initiation of postnatal glucose production [115].

Overall, the available evidence suggests that FABP4 could potentially be involved in linking maternal metabolic status, placental lipid metabolism, fetal growth, and neonatal metabolic adaptation. The significant postnatal increase in circulating FABP4 concentrations and its potential interaction with glucose-regulatory pathways suggest that FABP4 may play a role in the metabolic adaptation required for postnatal life. However, this requires further research.

## 4. Potential Clinical Implications

Increasing evidence suggests that maternal circulating FABP4 may also be associated with a range of maternal and fetal complications. A summary of the molecular mechanisms of FABP4 described in this review is presented in Table 1.

However, prospective longitudinal studies are needed to determine whether FABP4 concentrations have reliable predictive value for adverse pregnancy outcomes and to establish their potential clinical relevance in maternal-fetal medicine.

Clinical studies have demonstrated a positive association between circulating FABP4 concentrations and maternal BMI [2], with this relationship being particularly evident among overweight and obese women [34]. These findings suggest that maternal FABP4 concentrations may reflect the degree of adiposity during pregnancy and could potentially serve as a biomarker of maternal metabolic status and the severity of metabolic disturbances, including metabolic syndrome and GDM [34,116]. However, it should be emphasized once again that, at present, the use of FABP4 as a biomarker for predicting these conditions in clinical practice remains theoretical.

Moreover, FABP4 has been implicated as a molecular mediator of obesity-related myometrial dysfunction, suggesting that modulating FABP4-associated signaling pathways could represent a therapeutic strategy to improve labor outcomes in pregnancies affected by maternal obesity [40]. Nevertheless, it remains unclear whether FABP4 provides clinically relevant information beyond established measures of adiposity and metabolic dysfunction.

In an animal study, Dong et al. observed that treatment with the selective FABP4 inhibitor BMS309403 improved systemic insulin sensitivity in obese mice [117]. FABP4 inhibition has also been associated with lower circulating glucose concentrations and improved lipid metabolic profiles, suggesting a role in the modulation of metabolic disturbances associated with obesity and diabetes [48]. Furthermore, a novel class of dual FABP4/FABP5 inhibitors, including Compounds **1**–**3**, showed substantial inhibitory activity against FABP4 in vitro, with potency comparable to or greater than the reference compound BMS309403 [36].

Overall, these experimental findings support the hypothesis that FABP4 inhibition may influence glucose and lipid metabolism [118]. However, further evidence from human studies is needed before these findings can be translated into clinical practice. Importantly, the potential metabolic benefits of FABP4 inhibition need to be evaluated in the context of potential adverse effects, particularly those affecting the cardiovascular system. In this context, BMS309403 has been associated with reduced cardiac contractility in experimental studies, raising concerns about its potential cardiovascular safety [119]. Further preclinical and clinical studies are therefore needed to better characterize the safety profile of prolonged FABP4 inhibition, particularly in populations potentially vulnerable to cardiovascular complications, including pregnant women and individuals with pre-existing cardiovascular disease [119]. The association between elevated circulating FABP4 concentrations and both the development and metabolic characteristics of GDM raises the possibility that FABP4 in the future may have utility as a biomarker for identification of women at increased risk of GDM [60]. In a prospective longitudinal pilot clinical study in a Mexican population, FABP4 alone showed high apparent discriminative capacity for early identification of GDM at 10–14 weeks of gestation, with an area under the receiver operating characteristic curve (AUC) of 0.91 [120]. However, clinically validated and standardized FABP4 cutoff values for the diagnosis or prediction of GDM and other FABP4-associated metabolic disorders have not yet been established. Furthermore, FABP4 is unlikely to be implemented in routine clinical practice in the foreseeable future [121].

FABP4 has also been studied in relation to reproductive and placental complications, as well as maternal metabolic disorders. It has been suggested that FABP4 may be useful as a potential molecular marker in patients with RPL. However, the molecular mechanisms that may explain its role in early placental development and pregnancy progression remain unclear [93]. Specific m6A modification patterns or alterations in FTO and IGF2BP3 expression could serve as molecular indicators of impaired placental function. In particular, analyzing m6A-associated signatures in maternal plasma cell-free RNA may offer new opportunities to investigate placental dysfunction before clinical manifestations become apparent [91]. Combining epitranscriptomic profiles with placental transcriptomic data, clinical parameters, and other molecular biomarkers could further support the development of individualized approaches to pregnancy risk assessment [98]. However, introducing these findings into perinatal medicine will require mechanistic validation, standardized analytical methods, prospective clinical studies, and thorough evaluation of the safety of potential targeted interventions.

FABP4 may also help regulate placental vascular function and therefore may be involved in the pathophysiology of PE. Vascular endothelial growth factor (VEGF) is a major regulator of placental angiogenesis and vascular development, and FABP4 has been identified as a downstream effector of VEGF signaling in endothelial cells [122]. Experimental knockdown of FABP4 in endothelial cells attenuated VEGF-induced angiogenesis and cellular proliferation [122]. These findings suggest that FABP4 may participate in VEGF-dependent endothelial responses; however, the implications of this interaction for the development or prevention of PE remain uncertain. Given the proposed role of FABP4 in PE pathogenesis, clinical studies have investigated its potential as a predictive biomarker for PE. A case–control clinical study reported higher maternal FABP4 concentrations at 8–13 weeks of gestation in women who subsequently developed PE, suggesting that altered FABP4 concentrations may precede the clinical manifestation of the disease [68]. Although these findings suggest that FABP4 could serve as an early biomarker of PE, prospective studies are necessary to determine its predictive performance, reproducibility, and clinical utility.

FABP4 inhibition has also been proposed as a potential approach to reducing the risk of neonatal macrosomia. An in vitro study reported that treating trophoblast cells with BMS309403 reduced fatty acid production. Although these findings suggest that FABP4 inhibition may influence lipid metabolism in trophoblast cells, the implications for fetal growth remain unclear [68]. In particular, it is currently unclear whether the observed cellular effects would result in measurable changes in fetal growth or reduce the incidence of macrosomia in vivo. Therefore, the potential relationship between FABP4 activity, trophoblast lipid metabolism, and excessive fetal growth remains hypothetical and requires confirmation in appropriate experimental models [68]. At present, the clinical applicability of these findings is very limited, and further extensive research is needed before their translation into clinical practice can be considered. A summary of clinical studies investigating FABP4 in pregnancy complications described in this review is presented in Table 2.

## 5. Conclusions and Future Directions

Fatty acid-binding protein 4 (FABP4) is an adipocyte-derived signaling protein involved in regulating lipid metabolism, glucose homeostasis, and inflammatory responses. In adipose tissue, it facilitates intracellular fatty acid transport and contributes to metabolic pathways involved in lipid oxidation and storage. Increasing evidence indicates that alterations in circulating FABP4 concentrations are associated with several pregnancy-related conditions, including maternal obesity, GDM, hypertensive disorders of pregnancy, and RPL.

FABP4 may play three distinct but overlapping roles in pregnancy: as a potential biomarker, a pathogenic mediator, and a potential therapeutic target, although the evidence supporting these roles remains limited.

As a potential biomarker, circulating FABP4 has emerged as a candidate for the early identification of women at increased risk of GDM and hypertensive disorders of pregnancy. Nevertheless, its clinical utility remains unestablished, and prospective studies in large, well-characterized cohorts are needed to determine its predictive potential and incremental value beyond established maternal risk factors and currently available biomarkers.

As a pathogenic mediator, experimental studies suggest that FABP4 may contribute to insulin resistance, inflammation, endothelial dysfunction, placental lipid accumulation, mitochondrial impairment, and altered myometrial contractility. However, these mechanisms are supported predominantly by in vitro and animal studies, and a causal role in pregnancy complications remains unconfirmed. Moreover, FABP4 may exert beneficial physiological effects on placental development, lipid transport, and neonatal metabolic adaptation, highlighting its context-dependent activity.

Overall, the biological significance of these associations remains incompletely understood. It is unclear whether altered FABP4 concentrations primarily reflect underlying metabolic and vascular disturbances or whether FABP4 itself contributes to the molecular mechanisms involved in the development and progression of these complications. A more comprehensive understanding of FABP4 biology during pregnancy may help clarify its role in maternal metabolic adaptation, placental function, reproductive physiology, fetal development, and long-term offspring health.

As a potential therapeutic target, FABP4 inhibition has improved metabolic outcomes in experimental models, but its efficacy and safety during pregnancy remain unknown. Potential cardiovascular adverse effects and the physiological functions of FABP4 warrant particular consideration.

Accordingly, the therapeutic potential of targeting FABP4-related pathways in pregnancy-associated metabolic and vascular disorders remains under investigation, with current evidence derived predominantly from experimental studies. Future mechanistic and prospective clinical studies should further elucidate how FABP4 expression and circulating concentrations are regulated throughout pregnancy, clarify whether FABP4 acts as a causal mediator or primarily reflects underlying metabolic and vascular dysfunction, and assess whether targeting FABP4-related pathways could be a safe and clinically relevant strategy to improve pregnancy outcomes.

## Figures and Tables

**Table 1:** Summary of the molecular mechanisms of FABP4 described in this review.

Mechanistic Domain	Molecular Pathway or Target	Cellular or Metabolic Effect
Fatty acid binding and transport	Intracellular binding of long-chain fatty acids; transendothelial fatty acid transport	Facilitates lipid trafficking from lipid droplets and across capillary endothelium to metabolically active tissues
Lipolysis and non-classical secretion	Interaction with hormone-sensitive lipase; AC-PKA and GC-PKG pathways	Promotes lipolysis-associated fatty acid mobilization and signal-peptide-independent FABP4 release
PPARγ-dependent transcription	Nucleocytoplasmic shuttling, ligand delivery to PPARγ, and regulation of PPARγ abundance/activity	Modulates adipogenesis, lipid-responsive gene transcription, insulin sensitivity, and PPARγ degradation
Inflammatory signaling	MAPK/JNK-AP-1 and NF-κB pathways; TNF-α and IL-6 feedback	Amplifies pro-inflammatory cytokine production and metabolic inflammation
Macrophage and NK-cell regulation	JAK2/STAT2 and SIRT3-dependent pathways; interferon regulation	Favors M1 macrophage polarization, modulates reactive oxygen species generation, and may suppress NK-cell interferon production
Insulin and glucose homeostasis	Macrophage-adipocyte crosstalk; pancreatic β-cell signaling; glucagon-dependent hepatic pathways	Modulates insulin signaling and secretion, hepatic gluconeogenesis, glycogenolysis, and glucose production
Endothelial and angiogenic signaling	Suppression of eNOS; VEGF-FABP4 signaling	Reduces nitric oxide-dependent endothelial function while supporting VEGF-induced proliferation and angiogenesis
Mitochondrial metabolism and oxidative stress	Mitochondria-associated membrane integrity, β-oxidation, oxidative phosphorylation, ATP production, and SOD2	Excess FABP4 may promote lipid overload; insufficient FABP4 may impair respiration and antioxidant capacity
Epitranscriptomic regulation	m6A modification mediated by FTO and the reader IGF2BP3	Regulates FABP4 mRNA methylation, stability, and expression
Myometrial energetics	FABP4-dependent lipid accumulation and disruption of mitochondria-associated membranes	Reduces fatty acid utilization, ATP generation, and uterine smooth-muscle contractile capacity

AC-PKA, adenylyl cyclase-protein kinase A; AP-1, activator protein 1; ATP, adenosine triphosphate; eNOS, endothelial nitric oxide synthase; FABP4, fatty acid-binding protein 4; FTO, fat mass and obesity-associated protein; GC-PKG, guanylyl cyclase–protein kinase G; IGF2BP3, insulin-like growth factor 2 mRNA-binding protein 3; IL-6, interleukin 6; JAK2, Janus kinase 2; JNK, c-Jun N-terminal kinase; m6A, N6-methyladenosine; MAPK, mitogen-activated protein kinase; M1, classically activated macrophage phenotype; mRNA, messenger RNA; NF-κB, nuclear factor kappa B; NK-cell, natural killer cell; PKA, protein kinase A; PKG, protein kinase G; PPARγ, peroxisome proliferator-activated receptor gamma; SIRT3, sirtuin 3; SOD2, superoxide dismutase 2; STAT2, signal transducer and activator of transcription 2; TNF-α, tumor necrosis factor alpha; VEGF, vascular endothelial growth factor.

**Table 2:** Summary of clinical studies investigating FABP4 in pregnancy complications.

Pregnancy Complication	Number of Patient (Study Group/Control Group)	Gestational Age at Sampling (Weeks of Gestation)	Biological Material Analyzed	Main Findings	Reference [Number]
Maternal obesity	40/40	25–28	Serum ELISA	No statistical differences in FABP4 levels between the groups Significantly higher FABP4 levels in patients with BMI > 25 in GDM and control groups (*p* < 0.001) Significantly positive correlation between FABP4 levels and BMI in GDM and control groups (*p* < 0.001)	Vorobjova T, et al. [34]
GDM	30/30	24–28	Plasma ELISA	Significantly higher plasma FABP4 levels in the GDM than in control group (*p* < 0.0001)	Li, Y.Y. et al. [45]
GDM	12/12	After elective caesarean section	Placenta, Decidua, Serum ELISA; PCR	Significantly upregulated expression of FABP4 mRNA in the placenta and decidua from pregnant women with GDM (*p* < 0.05) Significantly higher total FABP4 levels in the placenta, decidua and serum in GDM group (*p* < 0.05) Increased serum expression of FABP4 in GDM group Increased serum circulating FABP4 in GDM group may originate from adipocytes and the placenta	Li, L. et al. [46]
GDM	135	First trimester 24–28	Plasma	In multivariate models comparing the second, third, and fourth quartiles against the first quartile, FABP4 levels in quartile 2, quartile 3, and quartile 4 were associated with the development of GDM Significant difference in the area under receiver operating characteristic curve between established risk factors alone and the addition of FABP4 concentrations (*p* = 0.03) Higher fatty FABP4 levels in the first trimester visit were associated with increased risk of GDM and might be useful in identifying women at risk for GDM for early prevention strategies.	Tu, W.J. et al. [59]
GDM	107/214 107/107	10–14, 15–26, all cases and controls 23–31, 33–39	Serum ELISA	At 10–14 GWs a model that included conventional GDM risk factors and FABP4, chemerin, soluble leptin receptor, and HMW-adiponectin improved the estimated prediction from 0.71 (95% CI 0.66 to 0.77) to 0.77 (95% CI 0.72 to 0.82) A panel of understudied adipokines including FABP4, chemerin, soluble leptin receptor may be implicated in the pathogenesis of GDM with significant associations detected approximately 10–18 weeks before typical GDM screening. No correlation between FABP4 levels and neonatal anthropometry.	Francis, E.C. et al. [61]
GDM	40/240	24–28	Serum ELISA	In GDM group higher levels of AFABP Pre-pregnancy BMI was the independent factor impacting serum AFABP levels in the second (β = 0.567, *p* = 0.004) and third trimesters (β = 0.619, *p* = 0.001). GDM was independently associated with AFABP levels in the second and third trimester (all *p* < 0.01)	Zhang, Y. et al. [63]
GDM	135/135	Before 14 24–28	Plasma ELISA	Similar FABP4 levels in GDM and control groups Compared with those in the lowest FABP4 tertile, women in the highest tertile of FABP4 levels in the first and second trimesters had 1.053 times and 1.447 times higher risk of developing GDM Elevated FABP4 levels in the first and second trimesters were associated with a higher level of insulin resistance and greater GDM risk, indicating FABP4 might predict women with high risk of developing GDM	Jin, C. et al. [63]
GDM (prediction)	200 19/15—final analyzes	11–14	Serum ELISA	FABP4 showed the greatest increase in the first trimester (4.9-fold, *p* = 0.0105) and the highest apparent discriminative performance (AUC = 0.91), which declined in the second and third trimesters. FABP4 emerged as a promising candidate biomarker for early GDM detection in early pregnancy	Arias-Covarrubias, S. et al. [120]
T1DM PE	710 TIDM	8–22 Median 14 Second trimester—median 26	Serum ELISA	FABP4 significantly elevated in early pregnancy and the second trimester (*p* < 0.001) in women who later developed preeclampsia. Addition of FABP4 to established risk factors significantly improved Net Reclassification FABP4 shows potential as a novel biomarker for preeclampsia prediction in women with type 1 diabetes.	Wotherspoon, A.C. et al. [76]
T1DM PE	23—who developed PE 24—who remained normotensive 19—healthy non-diabetic normotensive	12.4 ± 1.8 weeks; 21.7 ± 1.4 weeks; 31.4 ± 1.5 weeks.	Plasma ELISA	Higher FABP4 levels in PE than in control group in 21.7 ± 1.4 week and 31.4 ± 1.5 week of gestation, in both T1DM Progressive increase of FABP4 levels throughout pregnancy in all groups	Kelly, C. et al. [77]
PE	26—PE 27—control—nonpregnant 27—control—healthy pregnant	29–40	Serum ELISA	Serum AFABP was significantly higher in preeclampsia than non-pregnancy (*p* < 0.001) and normal pregnancy (*p* = 0.001) No significant difference in serum AFABP between mild and severe preeclampsia.	Shangguan, X. et al. [67]
PE	22—who developed PE 72—non-PE	8–13 24–28	Serum ELISA	Maternal serum FABP4 levels elevated before the clinical onset of preeclampsia, and this increase occurs independently of maternal body mass index. FABP4 was associated with the development of preeclampsia (adjusted odds ratio, 1.2; 95% *p* < 0.01).	Scifres, C.M. et al. [68]
PE	16/20	29.6 ± 4.4	Serum ELISA	Mean maternal AFABP concentrations were significantly elevated in PE as compared to controls Elevated FABP4 level in patients with BMI > 25 than BMI < 25 (*p* < 0.05) in PE group.	Fasshauer, M. et al. [70]
PE	60/60	early-onset group (*n* = 30, ≤34 weeks of gestation) late-onset group (*n* = 30, >34 weeks of gestation) 60 uncomplicated pregnancies (30 ≤ 34, and 30 > 34)	Serum ELISA Placenta PCR	Maternal serum FABP4 in early-onset PE group and in late-onset PE group, significantly elevated Mean placental FABP4 mRNA expression were significantly elevated in the early-onset PE group and late-onset PE group (no significant differences between two PE groups) Umbilical cord blood FABP4 levels significantly decreased in the early-onset PE group and late-onset PE group Umbilical cord blood FABP4 levels correlated negatively with maternal serum FABP4 level and placental FABP4 mRNA expression, but positively with neonatal weight. Increased FABP4 mRNA expression in placenta may contribute to high serum FABP4 level in women with PE.	Yan, J.Y. et al. [108]
RPL	28/37	Control 6.68 ± 0.97 RPL 7.52 ± 1.24	placental tissues PCR Western blot	FABP4 expression significantly downregulated in RPL villous tissues at both mRNA and protein levels (*p* = 0.02)	Dai, F. et al. [91]
GDM/Neonates	26/28	>37	Umbilical cord and maternal serum ELISA	Higher FABP4 levels in umbilical cord serum in GDM offspring Correlation umbilical cord FABP4 serum levels with the maternal serum FABP4 levels	Patro-Małysza, J. et al. [107]
LGA	770 samples were divided into three groups according to the infant birth weight as follows: low, <2500 g; normal, 2500–4000 g; and high >4000 g.	12–14 At delivery	Serum ELISA	The level of A-FABP in the serum of the mother at 12th–14th weeks of gestation is directly related to the birth weight of the future neonate Elevated maternal FABP4 concentrations have been associated with an increased risk of fetal macrosomia, defined as a birth weight greater than 4000 g	Ciborowski, M. et al. [109]
FGR	26 FGR pregnancies–without abnormal fetal Doppler 25 FGR pregnancies- abnormal fetal Doppler 32—controls	N/A	Serum ELISA	Significantly higher FABP-4 levels in the FGR cases with abnormal Doppler flow pattern group than in the FGR cases without abnormal Doppler flow pattern group and the control group (*p* < 0.001). A 1.35 ng/mL cut-off value of serum FABP-4 level could be used to discriminate FGR cases The optimal cut-off value of FABP-4 levels as an indicator for the diagnosis of FGR with abnormal Doppler flow pattern—1.76 ng/mL, FABP-4 is a powerful, reliable, and unique biomarker to diagnose FGR pregnancies	Bolluk G. et al. [111]
SGA	361	At delivery	Plasma ELISA	A negative correlation between AFABP and gestational age Higher AFABP levels in preterm infants compared with full-term infants (*p* < 0.01) Among full-term infants, AFABP levels in SGA infants were lower, compared with appropriate for gestational age and large for gestational age infants (*p* < 0.05).	Joung, K.E. et al. [113]

A-FABP, adipocyte fatty acid-binding protein; AGA, appropriate for gestational age; AUC, area under the curve; BMI, body mass index; CI, confidence interval; ELISA, enzyme linked immunosorbent assay; FABP4, fatty acid-binding protein; FGR, fetal growth restriction; GDM, gestational diabetes mellitus; LGA, large-for-gestational age; PE, preeclampsia; PCR, polymerase chain reaction; RPL, recurrent pregnancy loss; SGA, small-for-gestational age.

## Data Availability

No new data were created or analyzed in this study. Data sharing is not applicable to this article.
